# Searching for the Solution

**Published:** 1918-10-26

**Authors:** 


### MEDICAL TRAINING. WHAT IS IT TO BE?

### Searching for the Solution.

Before profound changes come, always there is a period of signs and portents, omens for the watcher. Any who qualified more than twenty years ago, and since has been in touch with student life and medical teachers, must have been aware of a stir of discontents, of feelings of a need for change in medical training and the examinations, no clear voice speaking, no definite scheme even outlined, but a muttering dissatisfaction. The muttering analysed was of two parts, a complaint of old graduates that the newly-qualified men were deficient in practical knowledge of clinical work though often full of the knowledge on which practical work must be based, a complaint of students and teachers that knowledge of too many subjects was required of the student and that so many examinations had to be prepared for, and faced at such frequent intervals, that student days were a time of cramming facts suited to examinations rather than a time for acquiring and digesting sound clinical experience.

There is reason for both complaints and much of truth. Surgery and medicine have made such way in the last twenty-five or thirty years, and the knowledge gained has been so well set forth, that now every student has to learn from text-books, and to pass examinations in, a far greater mass of matter than his predecessors of five-and-twenty years ago. More physiology, immensely more bacteriology, and a far higher standard of special subjects and special branches of surgery and medicine have to be acquired for examination purposes now than then. Probably the man qualified in the 'seventies and 'eighties left hospital with a greater practical knowledge of the work he expected to do in practice than the student of the twentieth century, but his ideas of what could be done were limited compared to the possibilities that face the younger generations. He was sound on common complaints and good at minor surgery and bandaging. He had spent much time in the casualty-room and many jolly evenings in the residents' rooms, from which, especially on Saturday nights, he sallied with the house surgeons or physicians on duty to see and help treat the victims of Bacchus and Venus that Saturday nights plentifully supplied. After all, most of us were destined to a life of minor surgery and common disease treatment, and during those jolly evenings we gained exactly the knowledge that would bring us our bread. We had not the same amount of reading to get through as those who are following in our footsteps, so we could afford the time for perhaps a desultory but certainly a practical picking-up of knowledge. Much useful experience fell to us, between the rubbers, the talk, and the bottled beer, when we went forth with the house surgeon at the call of the hospital porter. Now more time must be spent over books and in laboratories, and at special classes and demonstrations, and so there is less time for acquiring that knowledge of the ordinary and that knowledge of men and women that has been so valuable to many of us who are on the wane.

Of the old brigade some settled into a rut because it was not in them to do other. They were content with what they knew. Others, of livelier intelligence and keener curiosity, watched, experimented, learnt, and kept on learning. There is the point. The qualification is only the first step in the student's career. It shows only that the foundation of knowledge is laid, and on it the practitioner, student still, must build and build and build all through his life till his powers fail him. So he shall always be maturing and so he will be year by year a better man. It is on this point that the Insurance Act is doing so much harm to the profession of healing. Raw from the hospital, hundreds drop straightway into panel practice. They have at hand a vast amount of clinical material but no time to study it. Pile up the numbers, draft off the troublesome cases to the hospitals, and pocket all the shillings possible is too often the policy. He who steps not forward up the steep hill of knowledge glides backward. Patients seen and treated forty to the hour add nothing to the doctor's clinical acumen.

Sir George Newman in his memorandum has widely surveyed the position of medicine and has suggested changes in teaching and training. His suggestions may bear fruit and are very valuable. He sees that the medical man who would really be a master in his profession must be a student after he has got his place on the Register. That is beyond dispute. He sees the examination difficulty. So long as there are examinations to pass and that must be passed, most men will work for the examination and not for pure love of knowledge. This must be so. It is a question of economics. Fail to pass, and re-examination will cost so many pounds in addition to the cost of living whilst the three, six, or twelve months passes by. Yet without examination how is it possible to ascertain that the student, even the most diligent student, has acquired a good knowledge of any subject?

Sir George thinks that there is a tendency for the teaching of each subject to deal too entirely with the subject, so that the student does not see that each subject is part of a great whole; that great whole the knowledge of the art of healing. There is truth in the contention; but probably it is not the part of teachers and demonstrators of any subject to point out the co-relations of their subject with the other subjects of the whole. Their work is to teach their own subject, and to help the student to learn it. A man may be a most efficient teacher of biology, bacteriology, physiology, or other subjects, and yet have little or no acquaintance with diagnosis and the treatment of patients, so may have no grasp of the whole. The men who should co-ordinate the knowledge the students, unqualified or qualified, have acquired or are

**Medical Training. What is it to be**—(*continued*).

acquiring, are the lecturers, especially the lecturers on surgery and medicine. In most hospitals the only course of lectures of any value is the course that is given at 9 A.M., and that is only of value because it forces the student to overcome the weakness of the flesh, and to leave his bed at an unaired hour of the morning. There is little advantage in hearing a gentleman with a poor delivery set forth facts more plainly put in a text-book. Every student feels that most of the lectures he listens to are waste of time. This is not the fault of the lecturers. They are usually most competent surgeons or physicians on the staff of the hospital. It was on account of their skill they were appointed to the hospital staff, not because they were great speakers or teachers. A man may be an exceedingly good bedside teacher, and yet of the poorest as a lecturer.

But there are men capable of giving great addresses full of deep thought and full of stimulating ideas. If nine-tenths of the lectures in every medical school were allowed to lapse in favour of small classes and demonstrations and the few great lecturers were invited to visit schools in rotation, and give at each two or three lectures every session, such broad reviews as they would give would stimulate the audience, and help students of all degrees to digest and arrange the knowledge they had acquired in class-rooms, dissecting-rooms, wards, laboratories, and in private practice.

Maybe it would be well to confine pre-graduate examinations to the more ordinary branches of medicine and surgery, midwifery, and diseases of women, and to give diplomas in the special subjects such as diseases of the eye, the ear, the nose, and throat, and so forth, after qualification, and after post-graduate courses. The attempt to acquire in the course of the student career full knowledge of all the subjects that have become recognised as special subjects leads to a but crude assimilation of varied knowledge. It would be possible perhaps to devise a curriculum and a system of examination which should allow a man with safety to the public to be launched on a bread-winning career, and to hold out as an incentive to further studies hopes of post-graduate diplomas in special subjects for which he had abilities, and in which he took interest. Such schemes bristle with difficulties, of course; but if one could be devised which set the medical man on his career more practically able to diagnose the ordinary run of cases and more ambitious to acquire further knowledge, both the public and the profession would benefit.

